# Optimal Heat Exchanger Area Distribution and Low-Temperature Heat Sink Temperature for Power Optimization of an Endoreversible Space Carnot Cycle

**DOI:** 10.3390/e23101285

**Published:** 2021-09-30

**Authors:** Tan Wang, Yanlin Ge, Lingen Chen, Huijun Feng, Jiuyang Yu

**Affiliations:** 1 Institute of Thermal Science and Power Engineering, Wuhan Institute of Technology, Wuhan 430205, China; wangtan1013@126.com (T.W.); geyali9@hotmail.com (Y.G.); huijunfeng@139.com (H.F.); yjy@wit.edu.cn (J.Y.); 2 Hubei Provincial Engineering Technology Research Center of Green Chemical Equipment, Wuhan 430205, China; 3 School of Mechanical & Electrical Engineering, Wuhan Institute of Technology, Wuhan 430205, China

**Keywords:** endoreversible Carnot cycle for space, power output, area distribution, heat sink temperature, performance optimization, finite-time thermodynamics

## Abstract

Using finite-time thermodynamics, a model of an endoreversible Carnot cycle for a space power plant is established in this paper. The expressions of the cycle power output and thermal efficiency are derived. Using numerical calculations and taking the cycle power output as the optimization objective, the surface area distributions of three heat exchangers are optimized, and the maximum power output is obtained when the total heat transfer area of the three heat exchangers of the whole plant is fixed. Furthermore, the double-maximum power output is obtained by optimizing the temperature of a low-temperature heat sink. Finally, the influences of fixed plant parameters on the maximum power output performance are analyzed. The results show that there is an optimal temperature of the low-temperature heat sink and a couple of optimal area distributions that allow one to obtain the double-maximum power output. The results obtained have some guidelines for the design and optimization of actual space power plants.

## 1. Introduction

Carnot [1] found that the maximum thermal efficiency (TEF) of all thermodynamic cycles under ideal conditions is the Carnot efficiency, which provides the upper limit of TEF for heat engines working between the temperatures of hot- and cold-side heat reservoirs. In order to approach the actual process and reform and improve classical thermodynamics, some scholars [2,3,4] established the endoreversible Carnot heat engine (ECHE) model with only thermal resistance loss considered. The TEF limit of this model at maximum power output (POW) was obtained, which is the CA efficiency [4]. Andresen et al. [5] first proposed the concept of finite-time thermodynamics (FTT). Since then, many scholars have used this theory to study different thermodynamic processes and cycles, and FTT theory has made great developments [6,7,8,9,10,11,12,13,14,15,16,17,18,19,20,21,22,23,24,25,26,27,28,29,30,31,32,33,34,35].

Many scholars have studied the performance of the ECHE with FTT theory [36,37,38,39]. Yan [36] obtained the basic optimization relationship between the POW and TEF of the ECHE. Sun et al. [37,38] replaced the finite-time constraint with the finite-area constraint, took a specific PO as the optimization objective and obtained the relationship between the principle of the minimum heat transfer (HT) area and the area characteristics of the steady-flow heat engine. Schwalbe and Hoffmann [39] introduced stochastic thermodynamics into the study of performance optimization of the ECHE.

Compared with a ground-based power plant, a space power plant presents a series of novel features. For example, due to the relatively low temperature of the space environment, the waste heat generated by a low-temperature heat sink (LTHS) must be dissipated to the environment through a special radiator panel to increase the POW of the plant. Many scholars have studied space power plants with classical thermodynamics [40,41,42,43,44]. The mass and size of the heat exchangers (HEXs) of space power plants have major impacts on the feasibility of the devices. Therefore, many scholars have optimized the mass and size of the HEX as well as the performance of the entire space power plant. Barrett [40,41,42] studied the HEX model of a closed Brayton cycle (CBC) in nuclear space plants. Toro and Lior [43] analyzed the effects of the main operating parameters of the CBC for space power plants on the relationships among the POW and TEF and the radiator panel area ratio under different working fluid (WF) space conditions. Liu et al. [44] optimized the CBC for space power plants and found that the overall mass of the power plant could be reduced by optimizing the core parameters of the plant components.

Some scholars have also studied space power plants with FTT theory [45,46,47,48,49]. References [45,46,47,48,49] established simple and regenerative CBC models in space nuclear plants and applied the thermal conductances of the HEXs to predict the energy conversion performance and analyze the effects of thermal conductances on the performances of the plants.

Based on the endoreversible Carnot cycle model established in References [2,3,4], considering a radiator panel between the LTHS and the relatively low temperature of a space environment to dissipate waste heat to space, a model of an endoreversible Carnot cycle for space is established in this paper. FTT theory is applied to analyze this model. General relationships between POW and TEF and the temperature of the LTHS are obtained. Taking the cycle POW as the optimization objective, the surface area distributions of the HEXs are optimized when the total area of HEXs of the whole plant is fixed, and the maximum POW is obtained. Furthermore, the double-maximum POW is obtained by optimizing the temperature of the LTHS. There are optimal temperatures of the LTHS and a couple of optimum area distributions, which lead to the double-maximum POW. Such temperature and area distribution conditions ensure the future design of a plant conversion system that aligns better performances and compactness. Finally, the influences of fixed plant parameters on the maximum POW performance are analyzed.

## 2. Cycle Model and Performance Indicators

**Figure 1:**
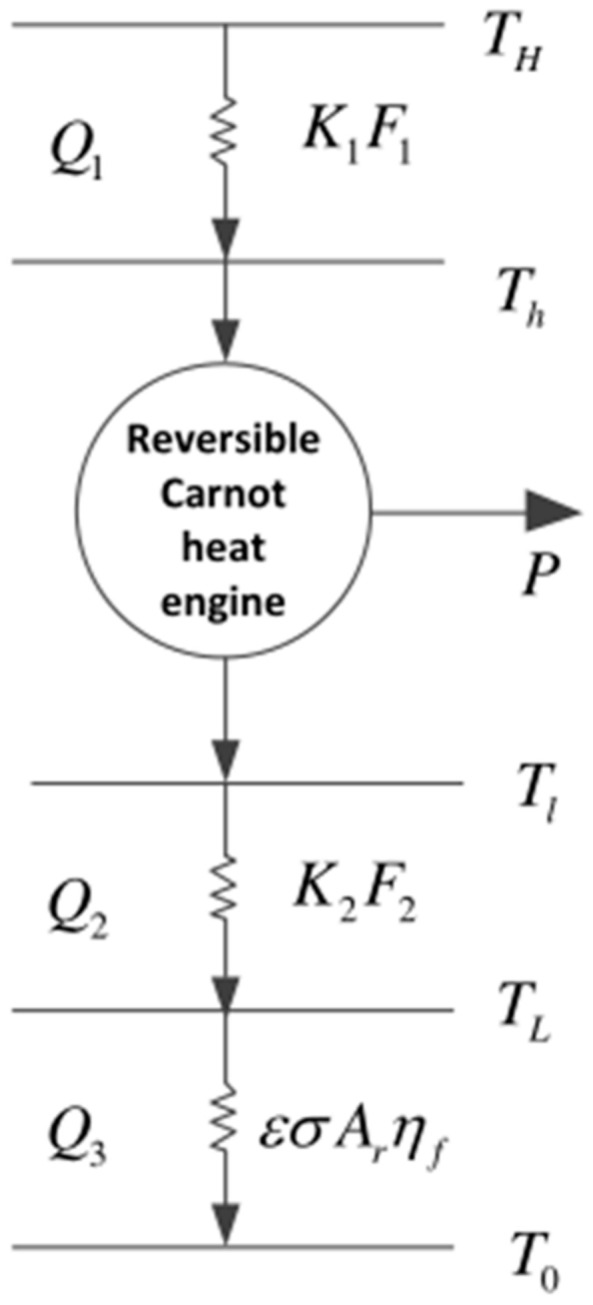
Model of Carnot cycle for space plant.

**Figure 2:**
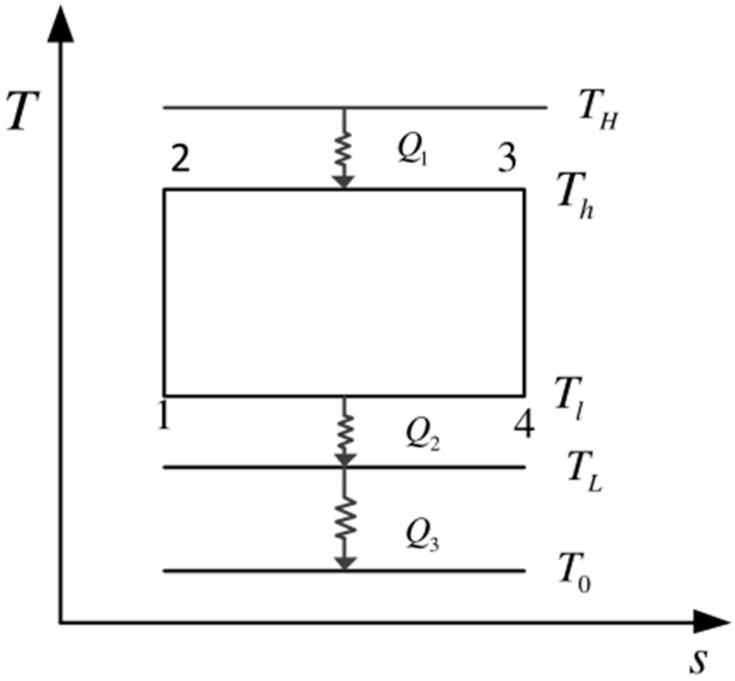
*T*-*s* Diagram of Carnot cycle for space.

Figure 1 shows an endoreversible Carnot cycle model for a space plant. Figure 2 shows its *T*-*s* diagram. In the figures, processes 1→2 and 3→4 are two adiabatic processes, and 2→3 and 4→1 are two isothermal processes. The actual device is simplified into a Carnot cycle, but the power plant is different from the ground-based Carnot cycle. The power plant uses HEXs between the WF and the heat reservoirs (the heat absorption and heat release processes of the WF are completed by the hot HEX and the cold HEX, respectively), and it is also necessary to use a radiator panel between the LTHS and the space environment to dissipate waste heat to space. $T_{H}$ and $T_{L}$ are the temperatures of the high- and low-temperature heat reservoirs, and $T_{h}$ and $T_{l}$ are the corresponding working temperatures of the WF.

Assuming that the heat transfer (HT) between the heat reservoir and the WF obeys Newton HT law, the heat flux rates are, respectively,

(1) $$Q_{1}=K_{1}F_{1}\left(T_{H}-T_{h}\right)$$

(2) $$Q_{2}=K_{2}F_{2}\left(T_{l}-T_{L}\right)$$

The radiator panel radiates the heat from the cold HEX to the space environment. According to Reference [44], the heat flux rate of the radiation HT is

(3) $$Q_{3}=\sigma \varepsilon A_{r}\eta_{f}\left(T_{L}^{4}-T_{0}^{4}\right)$$

where $K_{1}$ ($K_{2}$) is the HT coefficient of the hot (cold) HEX, $F_{1}$ ($F_{2}$) is the surface area of the hot (cold) HEX, ε is the emissivity of the radiator, $A_{r}$ is the area of the radiation panel surface, σ is the Boltzmann constant, $\eta_{f}$ is the fin efficiency, and $T_{0}$ is the ambient temperature.

According to the endoreversible condition and the first law of thermodynamics, one has

(4) $$P=Q_{1}-Q_{2}$$

(5) $$Q_{2}=Q_{3}=\frac{T_{l}}{T_{h}}Q_{1}$$

From Equations (4) and (5), one has

(6) $$P=Q_{1}-Q_{2}=Q_{1}\left(1-\frac{T_{l}}{T_{h}}\right)$$

From Equations (1)–(4), one has

(7) $$T_{l}=\frac{\sigma \varepsilon A_{r}\eta_{f}\left(T_{L}^{4}-T_{0}^{4}\right)}{K_{2}F_{2}}+T_{L}$$

(8) $$T_{h}=\frac{K_{1}F_{1}T_{H}\sigma \varepsilon A_{r}\eta_{f}\left(T_{L}^{4}-T_{0}^{4}\right)+K_{1}F_{1}K_{2}F_{2}T_{H}T_{L}}{\sigma \varepsilon A_{r}\eta_{f}\left(K_{1}F_{1}+K_{2}F_{2}\right)\left(T_{L}^{4}-T_{0}^{4}\right)+K_{1}F_{1}K_{2}F_{2}T_{L}}$$

From Equations (7) and (8), one has

(9) $$\frac{T_{l}}{T_{h}}=\frac{\sigma \varepsilon A_{r}\eta_{f}\left(K_{1}F_{1}+K_{2}F_{2}\right)\left(T_{L}^{4}-T_{0}^{4}\right)+K_{1}F_{1}K_{2}F_{2}T_{L}}{K_{1}F_{1}K_{2}F_{2}T_{H}}$$

Substituting Equations (1), (7) and (8) into Equation (5), one has

(10) $$\begin{array}{c} P=K_{1}F_{1}\left(T_{H}-\frac{K_{1}F_{1}T_{H}\sigma \varepsilon A_{r}\eta_{f}\left(T_{L}^{4}-T_{0}^{4}\right)+K_{1}F_{1}K_{2}F_{2}T_{H}T_{L}}{\sigma \varepsilon A_{r}\eta_{f}\left(K_{1}F_{1}+K_{2}F_{2}\right)\left(T_{L}^{4}-T_{0}^{4}\right)+K_{1}F_{1}K_{2}F_{2}T_{L}}\right)\\ \left(1-\frac{\sigma \varepsilon A_{r}\eta_{f}\left(K_{1}F_{1}+K_{2}F_{2}\right)\left(T_{L}^{4}-T_{0}^{4}\right)+K_{1}F_{1}K_{2}F_{2}T_{L}}{K_{1}F_{1}K_{2}F_{2}T_{H}}\right) \end{array}$$

The TEF of the cycle is defined by

(11) $$\eta =P/Q_{1}$$

Substituting Equations (1), (8) and (10) into Equation (11), one has

(12) $$\eta =\left(1-\frac{\sigma \varepsilon A_{r}\eta_{f}\left(K_{1}F_{1}+K_{2}F_{2}\right)\left(T_{L}^{4}-T_{0}^{4}\right)+K_{1}F_{1}K_{2}F_{2}T_{L}}{K_{1}F_{1}K_{2}F_{2}T_{H}}\right)$$

## 3. Power Optimization

In the actual design process, the total HT area $F_{T}$ ($F_{T}=F_{1}+F_{2}+F_{3}$) of the HEXs is finite. When $F_{T}$ is fixed, the area of each HE should be reasonably distributed to improve the performance of the power plant.

For the fixed total HT area ($F_{T}$) of the HEXs, the area distribution is defined as

(13) $$f_{i}=F_{i}/F_{T}\;(i=1,2,3)$$

So, the hot HEX area distribution ($f_{1}$) and the cold HEX area distribution ($f_{2}$) are, respectively,

(14) $$f_{1}=F_{1}/F_{T},\;f_{2}=F_{2}/F_{T}$$

The radiator panel area distribution is

(15) $$F_{3}=(1-f_{1}-f_{2})F_{T}$$

The area distribution should satisfy the following relationship:

(16) $$\sum f_{i}=1,\;0<f_{i}<1$$

Taking the cycle POW as the optimization objective, the area distributions of the three HEXs can be optimized, and the maximum POW can be obtained when the total HT area of the HEXs of the whole plant is fixed. Furthermore, the double-maximum POW can be obtained by optimizing the temperature of the LTHS. In this paper, the optimization results of the POW are numerically calculated. According to References [37,38,46], the following parameters are determined: $\sigma =5.67\times 10^{-8}{\;W/(m}^{2}\cdot K^{4})$, $\eta_{f}=0.9$, $F_{T}=20{\sim 40\;m}^{2}$, $K_{1}F_{T}=K_{2}F_{T}=2\sim 6\;W/K$, ε=0.9, $T_{0}=180\;K\sim 220\;K$ and $T_{H}=1050\;K\sim 1250\;K$.

**Figure 3:**
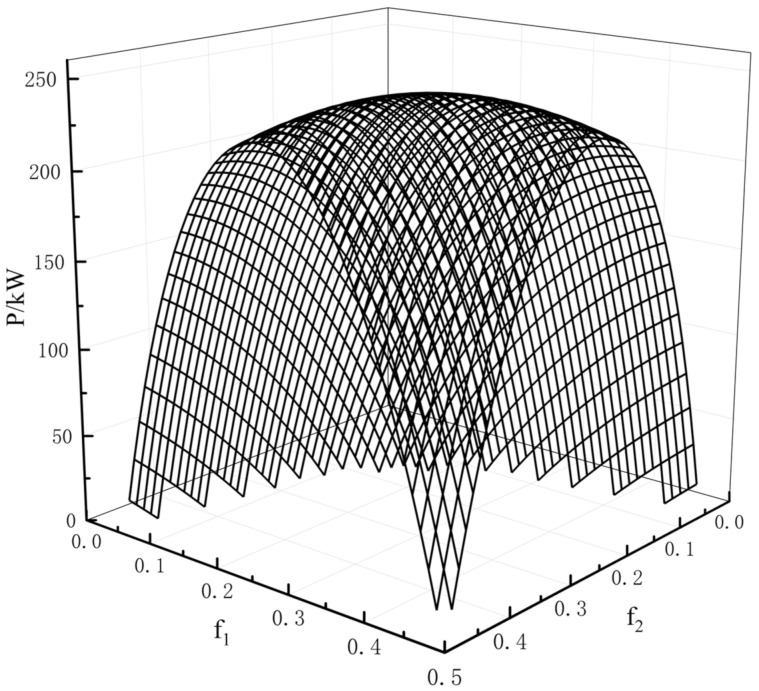
Relation of P versus $f_{1}$ and $f_{2}$.

**Figure 4:**
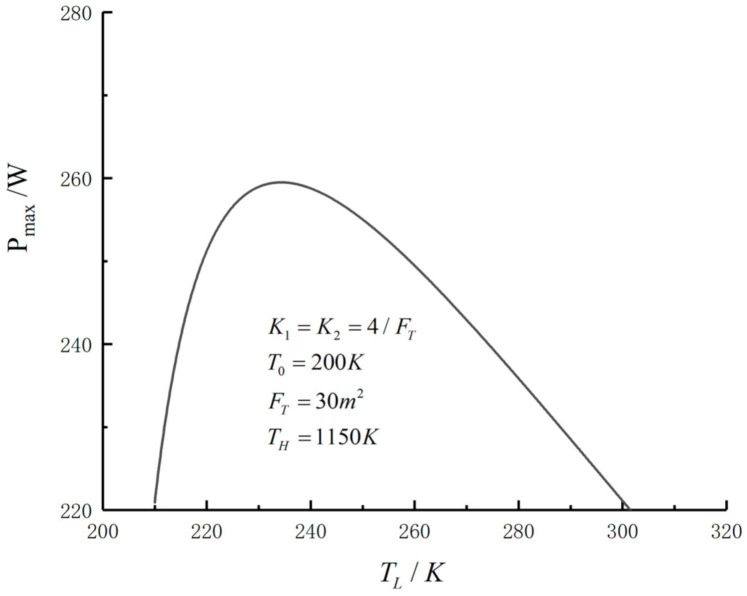
Relation of $P_{\max}$ versus $T_{L}$.

Figure 3 shows a three-dimensional relationship among the POW and the hot HEX area distribution $f_{1}$ and the cold HEX area distribution $f_{2}$ when $F_{T}{\;=\;30\;m}^{2}$, $T_{H}=1150\;K$, $T_{0}=200\;K$ and $K_{1}=K_{2}=4/F_{T}$. The figure shows that there is a couple of optimal distributions ($f_{1_{\mathrm{opt}}}$ and $f_{2_{\mathrm{opt}}}$) for the fixed $F_{T}$ and $T_{L}$, which result in the maximum POW ($P_{\max}$). Figure 4 shows the relationship between the maximum POW and the temperature of the LTHS when the area distributions are the optimal values. One can see that $P_{\max}-T_{L}$ is a parabolic-like one, and there is an optimal $T_{L}{}_{{}_{opt}}$, which will lead to the double-maximum POW ($P_{\max,\max}$). When $T_{L}$ is fixed, there exists a couple of area distributions that result in the maximum POW ($P_{\max}$), and when the area distribution is fixed, there is an optimal $T_{L}{}_{{}_{opt}}$, which also results in $P_{\max}$. So, there is an optimal $T_{L}{}_{{}_{opt}}$ and a couple of optimum area distributions that lead to the double-maximum POW ($P_{\max,\max}$).

**Figure 5:**
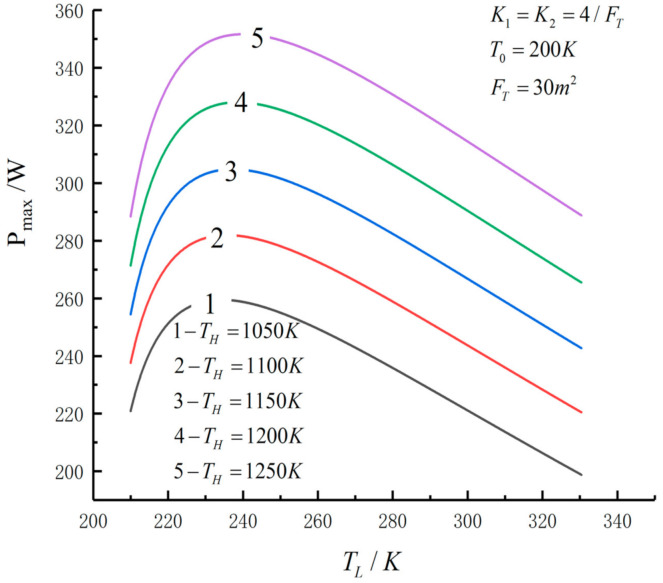
$P_{\max}$ versus $T_{L}$ under different $T_{H}$.

**Figure 6:**
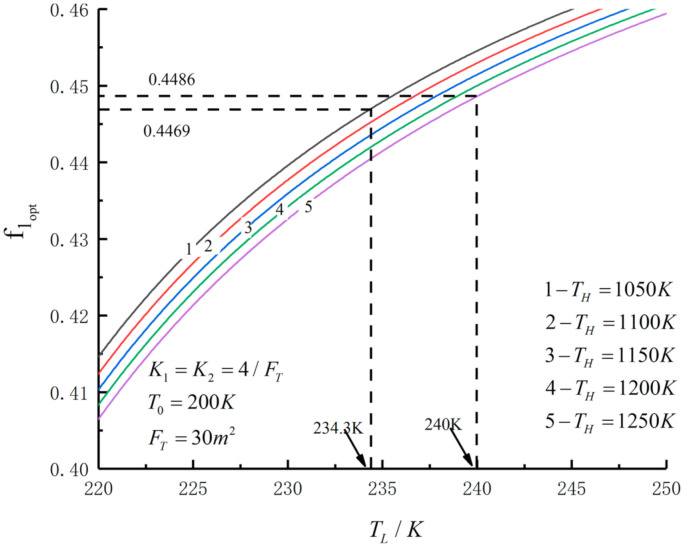
$P_{\max}$ versus $T_{L}$ under different $T_{H}$.

**Figure 7:**
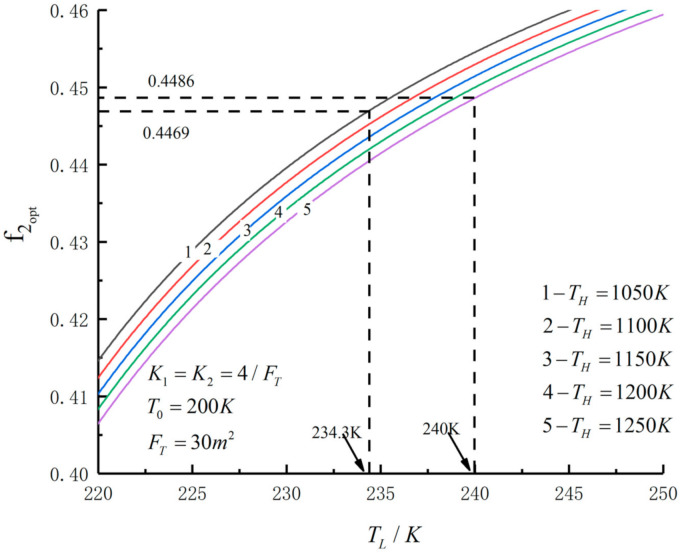
$P_{\max}$ versus $T_{L}$ under different $T_{H}$.

**Figure 8:**
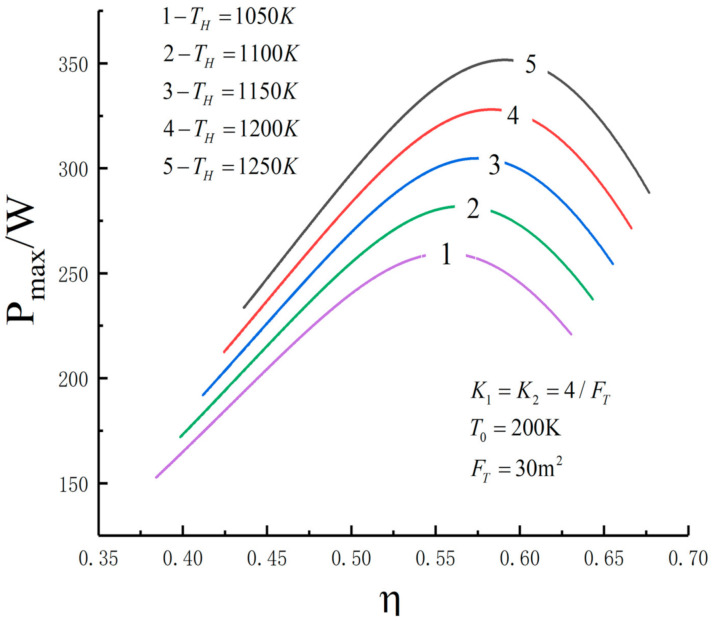
$P_{\max}$ versus η under different $T_{H}$.

**Figure 9:**
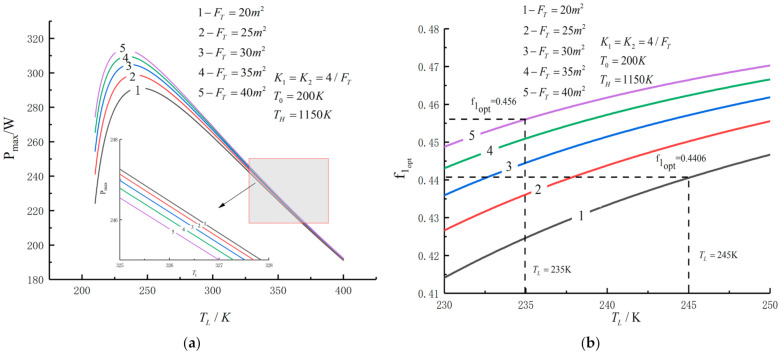

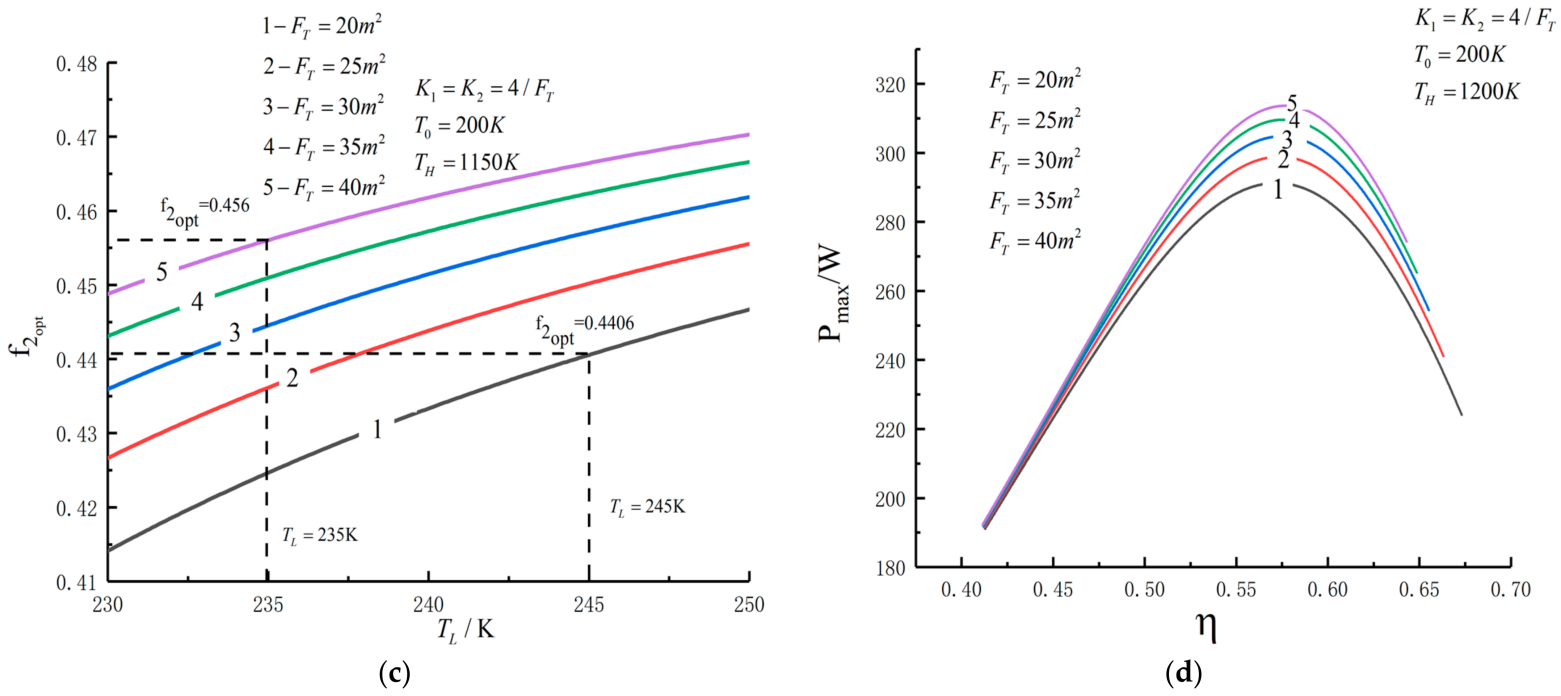
(**a**) $P_{\max}$, (**b**) $f_{1_{opt}}$ and (**c**) $f_{2_{opt}}$ versus $T_{L}$ under different $F_{T}$; (**d**) $P_{\max}$ versus η under different $F_{T}$.

**Figure 10:**
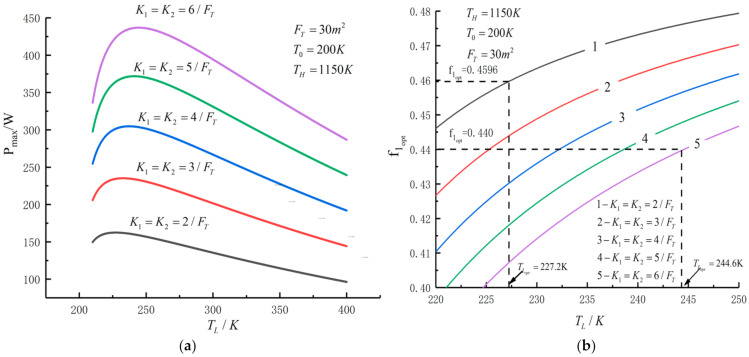

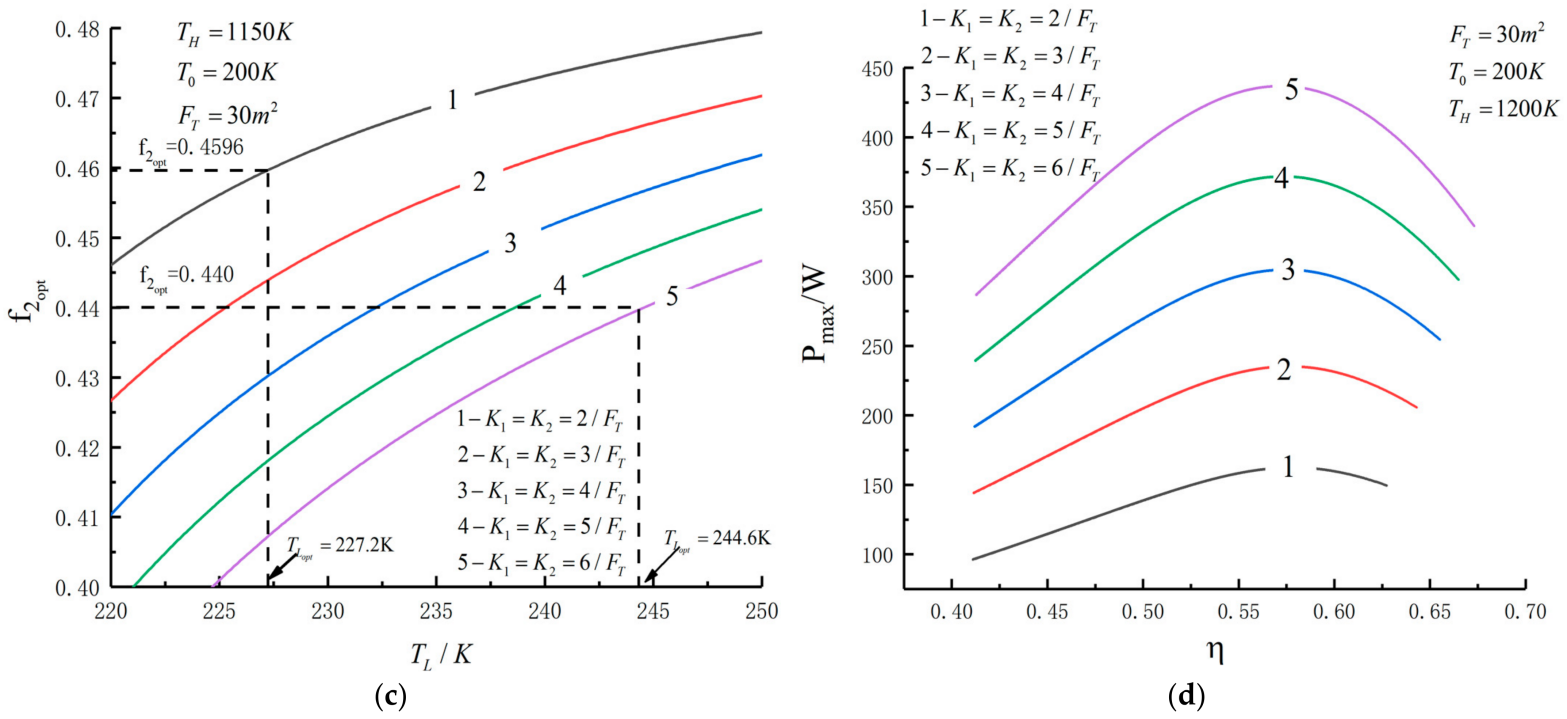
(**a**) $P_{\max}$, (**b**) $f_{1_{opt}}$ and (**c**) $f_{2_{opt}}$ versus $T_{L}$ under different $K_{1}$ and $K_{2}$; (**d**) $P_{\max}$ versus η under different $K_{1}$ and $K_{2}$.

**Figure 11:**
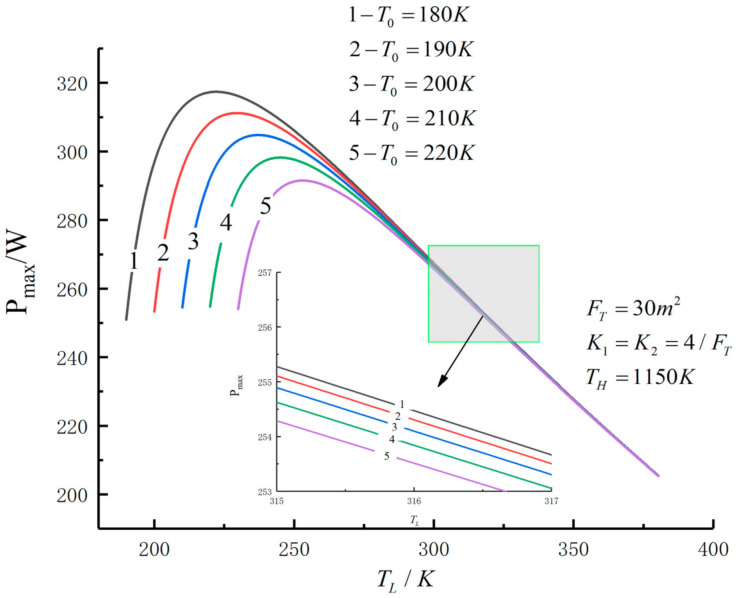
$P_{\max}$ versus $T_{L}$ under different $T_{0}$.

**Figure 12:**
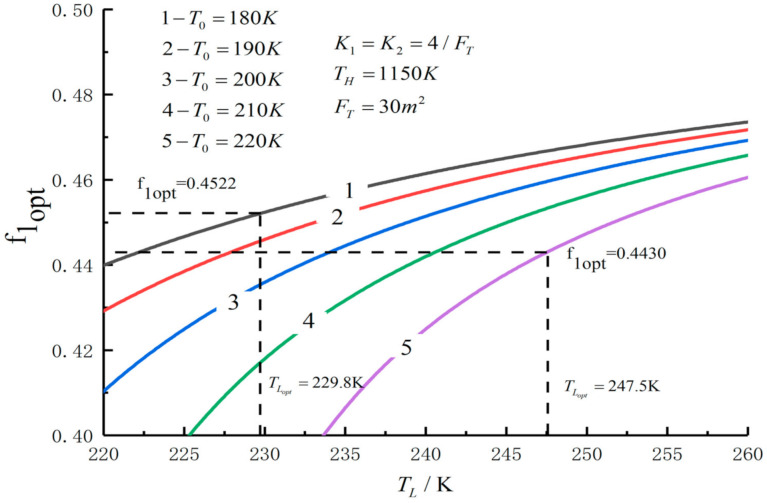
$f_{1_{opt}}$ versus $T_{L}$ under different $T_{0}$.

**Figure 13:**
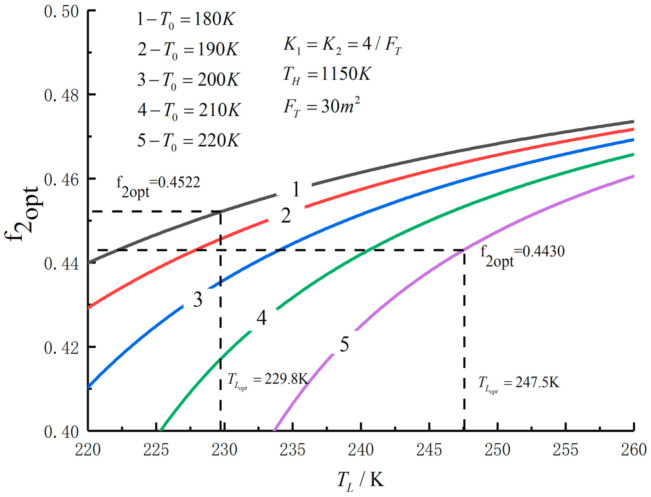
$f_{2_{opt}}$ versus $T_{L}$ under different $T_{0}$.

**Figure 14:**
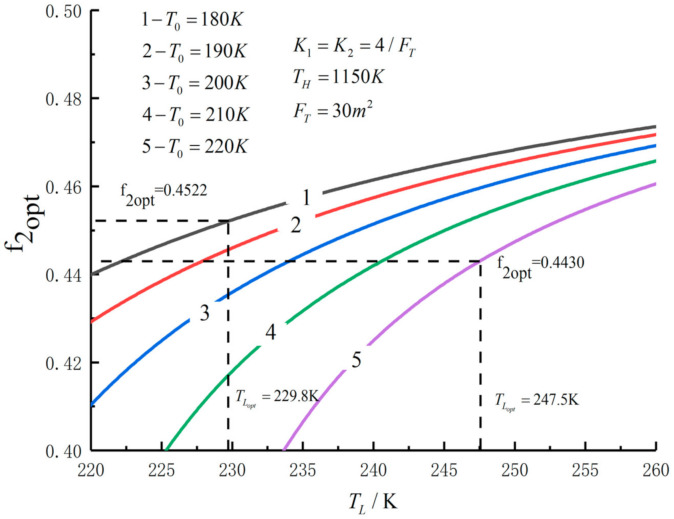
$P_{\max}$ versus η under different $T_{0}$.

Figure 5, Figure 6, Figure 7, Figure 8, Figure 9, Figure 10, Figure 11, Figure 12, Figure 13 and Figure 14 show the effects of $T_{H}$, $F_{T}$, $K_{1}$, $K_{2}$ and $T_{0}$ on $P_{\max}-T_{L}$, $f_{1_{opt}}-T_{L}$, $f_{2_{opt}}-T_{L}$ and $P_{\max}-\eta$ characteristics. $T_{H}$, $F_{T}$, $K_{1}$, $K_{2}$ and $T_{0}$ are fixed parameters; $T_{H}$ and $T_{0}$ depend on the external environment; and $K_{1}$, $K_{2}$ and $F_{T}$ depend on the material properties of the HEXs and the technology. The major point of this paper is to optimize the area distribution of the three HEXs for the fixed total area of the HEXs, thereby optimizing the temperature of the working fluid to optimize the cycle performance, and to analyze the effects of fixed parameters on the cycle performance.

**Figure 15:**
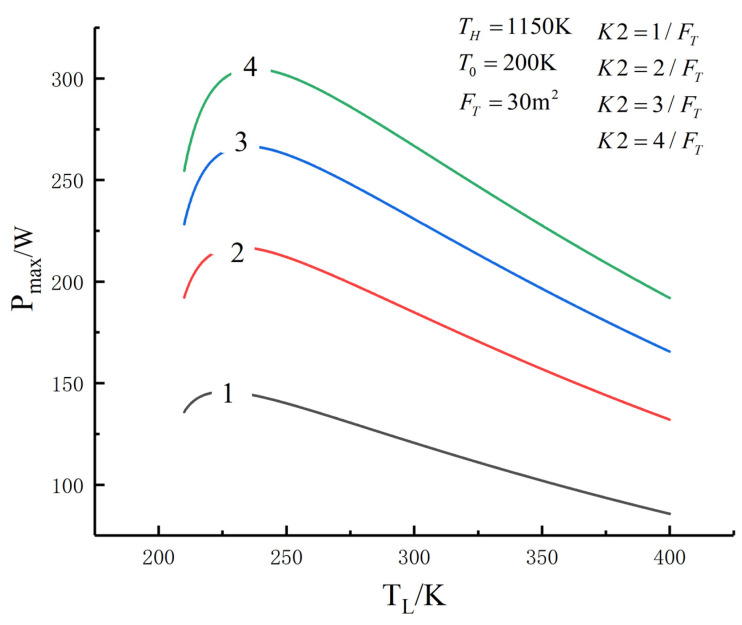
$P_{\max}$ versus $T_{L}$ under different $K_{2}$.

**Figure 16:**
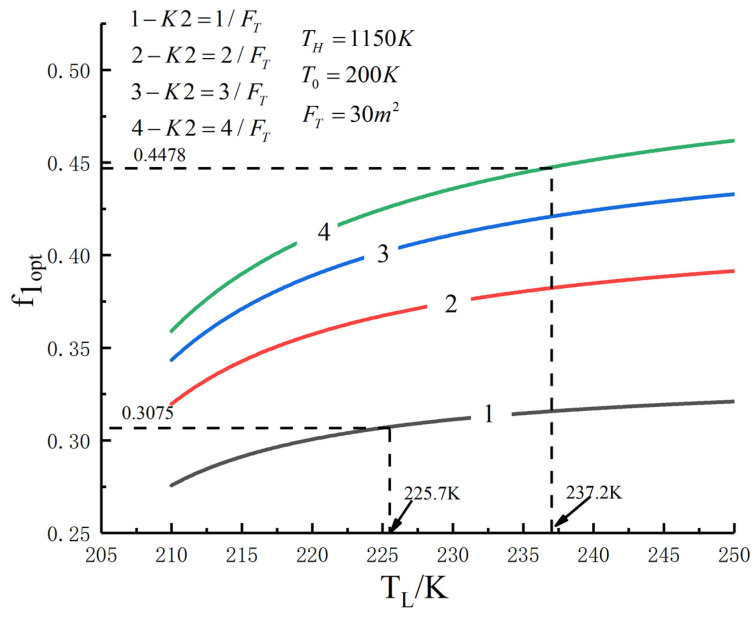
$f_{1_{opt}}$ versus $T_{L}$ under different $K_{2}$.

**Figure 17:**
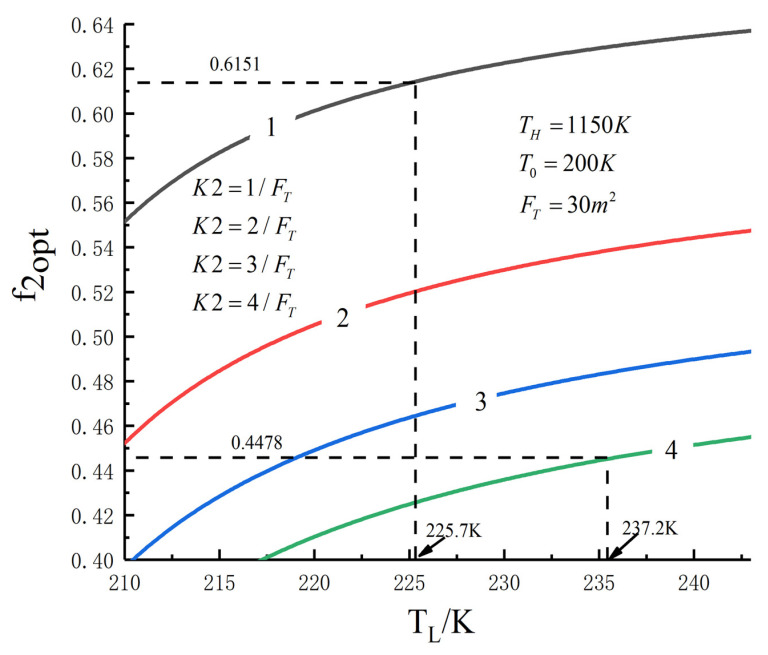
$f_{2_{opt}}$ versus $T_{L}$ under different $K_{2}$.

**Figure 18:**
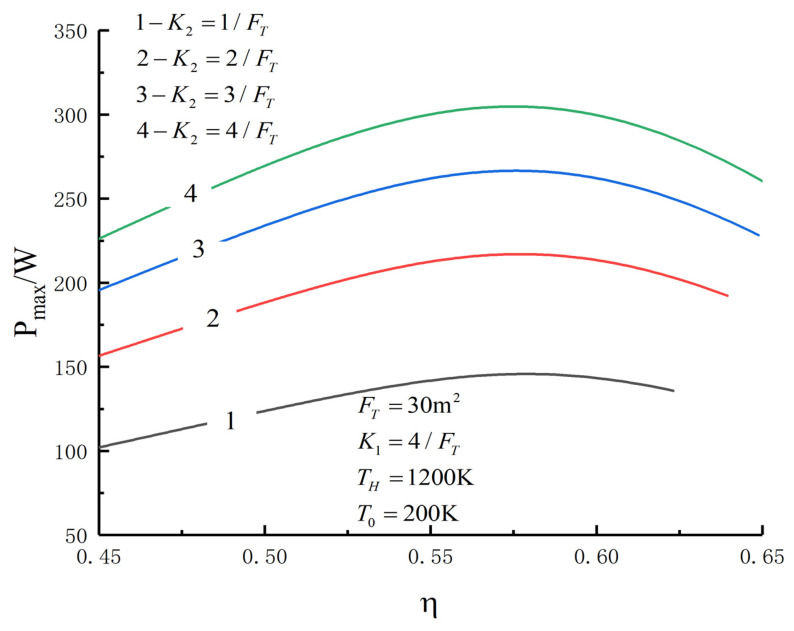
$P_{\max}$ versus η under different $K_{2}$.

One can see that the optimal area distributions of the HEXs increase with an increase in $T_{L}$; the curve of $P_{\max}-\eta$ is a parabolic-like one. The corresponding TEF under the double-maximum POW is $\eta_{P}{}_{{}_{\max}}$. Figure 15, Figure 16, Figure 17 and Figure 18 show the effects of $K_{2}$ on $P_{\max}-T_{L}$, $f_{1_{opt}}-T_{L}$, $f_{2_{opt}}-T_{L}$ and $P_{\max}-\eta$ characteristics when $K_{1}\neq K_{2}$.

Figure 5, Figure 6, Figure 7 and Figure 8 show the influence of $T_{H}$ on the relationships between $P_{\max}-T_{L}$, $f_{1_{opt}}-T_{L}$, $f_{2_{opt}}-T_{L}$ and $P_{\max}-\eta$. With an increase in $T_{H}$, $P_{\max,\max}$, $\eta_{P}{}_{{}_{\max}}$, $f_{1_{opt}}$, $f_{2_{opt}}$ and $T_{L_{opt}}$ will increase. When $T_{H}$ increases from 1050 K to 1250 K, $P_{\max,\max}$ increases from 259.50 W to 351.65 W and increases by 35.5%, $\eta_{P}{}_{{}_{\max}}$ increases from 0.556 to 0.591 and increases by 6.3%, $f_{1_{opt}}$ and $f_{2_{opt}}$ increase from 0.4469 to 0.4486 and increase by 0.38% and $T_{L_{opt}}$ increases from 234.3 K to 240 K and increases by 2.43%. When $F_{T}{\;=30\;m}^{2}$, $T_{H}=1250\;K$, $T_{0}=200\;K$ and $K_{1}=K_{2}=4/F_{T}$, the Novikov–Curzon–Ahlborn efficiency is 0.60 according to equation $\eta_{CA}=1-\sqrt{T_{L}/T_{H}}$, which was derived from References [2,3,4]. The TEF at the double maximum POW is 0.591 obtained herein. The Carnot efficiency is 0.84 according to equation $\eta_{C}=1-\left(T_{L}/T_{H}\right)$, which was derived from Reference [1]. The maximum TEF is 0.84. One can see that the TEF at the double-maximum POW is close to CA efficiency, and the maximum TEF and the Carnot efficiency are the same.

Figure 9 shows the influences of $F_{T}$ on the relationships between $P_{\max}-T_{L}$, $f_{1_{opt}}-T_{L}$, $f_{2_{opt}}-T_{L}$ and $P_{\max}-\eta$. With an increase in $F_{T}$, $P_{\max,\max}$, $f_{1}{}_{{}_{opt}}$, $f_{2}{}_{{}_{opt}}$ and $\eta_{P_{\max}}$ will increase, while $T_{L_{opt}}$ will decrease. When $F_{T}$ increases from $20\;m^{2}$ to $40\;m^{2}$, $P_{\max}$ increases from 291.24 W to 313.46 W and increases by 7.6%, $f_{1_{opt}}$ and $f_{2_{opt}}$ increase from 0.4406 to 0.4560 and increase by 3.5%, $\eta_{P_{\max}}$ increases from 0.572 to 0.576 and increases by 0.7% and $T_{L_{opt}}$ decreases from 245 K to 235 K and decreases by 0.4%.

Figure 10 shows the influences of $K_{1}$ and $K_{2}$ on the relationships between $P_{\max}-T_{L}$, $f_{1_{opt}}-T_{L}$, $f_{2_{opt}}-T_{L}$ and $P_{\max}-\eta$. With an increase in $K_{1}$ and $K_{2}$, $P_{\max,\max}$ and $T_{L_{opt}}$ will increase, while $f_{1_{opt}}$, $f_{2_{opt}}$ and $\eta_{P_{{}_{\max}}}$ will decrease. When $K_{1}$ and $K_{2}$ increase from $2/F_{T}$ to $6/F_{T}$, $P_{\max,\max}$ increases from 162.46 W to 436.87 W and increases by 169%, $f_{1_{opt}}$ and $f_{2_{opt}}$ decrease from 0.4596 to 0.440 and decrease by 4.26%, $\eta_{\max}$ decreases from 0.578 to 0.571 and decreases by 1.2% and $T_{L_{opt}}$ increases from 227.2 K to 244.6 K and increases by 7.66%.

Figure 11, Figure 12, Figure 13 and Figure 14 show the influences of $T_{0}$ on the relationships between $P_{\max}-T_{L}$, $f_{1_{opt}}-T_{L}$, $f_{2_{opt}}-T_{L}$ and $P_{\max}-\eta$. With a decrease in $T_{0}$, $P_{\max}$, $\eta_{\max}$ and $T_{L_{opt}}$ will increase, while $f_{1_{opt}}$ and $f_{2_{opt}}$ will decrease. When $T_{0}$ decreases from 220 K to 180 K, $P_{\max,\max}$ increases from 291.52 W to 317.40 W and increases by 8.9%, $f_{1_{opt}}$ and $f_{2_{opt}}$ decrease from 0.4522 to 0.4430 and decrease by 2%, $\eta_{P_{\max}}$ increases from 0.557 to 0.593 and increases by 6.5% and $T_{L_{opt}}$ increases from 229.8 K to 247.5 K and increases by 7.7%.

Figure 15, Figure 16, Figure 17 and Figure 18 show the influences of $K_{2}$ on the relationships between $P_{\max}-T_{L}$, $f_{1_{opt}}-T_{L}$, $f_{2_{opt}}-T_{L}$ and $P_{\max}-\eta$ when $K_{1}\neq K_{2}$. With an increase in $K_{2}$, $P_{\max}$, $f_{1_{opt}}$ and $T_{L_{opt}}$ will increase, while $f_{2_{opt}}$ will increase. When $K_{2}$ increases from $1/F_{T}$ to $4/F_{T}$, $P_{\max}$ increases from 145.76 W to 304.79 W and increases by 109.1%, $f_{1_{opt}}$ increases from 0.3075 to 0.4478 and increases by 55.4%, $f_{2_{opt}}$ decreases from 0.6151 to 0.4478 and decreases by 27.2% and $T_{L_{opt}}$ increases from 225.7 K to 237.2 K and increases by 5.1%.

## 4. About FTT

Some ones have some controversies about FTT. It is necessary to discuss it further. As Tang et al. [50] pointed out the following about FTT:

*“FTT is the further extension of conventional irreversible thermodynamics. The cycle model established by Curzon and Ahlborn [4] was a reciprocating Carnot cycle, and the finite time was its major feature. Therefore, such problems of extremal of thermodynamic processes were first named as FTT by Andresen et al [5] and as Optimization Thermodynamics or Optimal Control in Problems of Extremals of Irreversible Thermodynamic Processes by Orlov and Rudenko [51]. When the research object was extended from reciprocating devices characterized by finite-time to the steady state flow devices characterized by finite size, one releases that the physical property of the problems is the heat transfer owing to temperature deference. Therefore, Grazzini [52] termed it as Finite Temperature Difference Thermodynamics, and Lu [53] termed it as Finite Surface Thermodynamics. In fact, the works performed by Moutier [54] and Novikov [2] were also steady state flow device models. While Bejan introduced the effect of temperature difference heat transfer on the total entropy generation of the systems, taken the entropy generation minimization as the optimization objective for designing thermodynamic processes and devices, and termed as “Entropy Generation Minimization” or “Thermodynamic Optimization” [55,56]. For the steady state flow device models, Feidt [15,57,58,59,60,61,62,63,64,65,66] termed it as Finite Physical Dimensions Thermodynamics (FPDT). The model established here in is closer to FPDT. For both reciprocating model and steady state flow model, the suitable name may be thermodynamics of finite size devices and finite time processes, as Bejan termed [55,56].”*

Muschik and Hoffmann [67] studied the connection between the endoreversible reciprocating model of FTT and the actual irreversible model. According to the idiomatic usage, the theory is termed as FTT in this paper.

## 5. Conclusions

Using FTT theory, a model of an endoreversible Carnot cycle for space plants is established in this paper. The expressions of the cycle POW and TEF are derived. The influences of various design parameters of the plant on the maximum POW performance are analyzed by numerical examples. The results obtained show the following:

(1) The relationships between $P_{\max}-T_{L}$ and $P_{\max}-\eta$ are parabolic-like ones. When the temperature of the LTHS is fixed, there are a couple of area distributions that allow one to obtain the maximum POW. At the same time, when the area distributions are fixed, there is an optimal temperature of the LTHS that allows one to obtain another maximum POW. So, there is an optimal temperature of the LTHS and a couple of optimal area distributions that allow one to obtain the double-maximum POW.
(2) The double-maximum POW, the corresponding TEF under the double-maximum PO, the optimal area distributions and the optimal temperature of the LTHS increase with an increase in the temperature of the high-temperature heat sink. With a decrease in the space environment, the double-maximum POW, the corresponding TEF under the double-maximum POW and optimal the temperature of the LTHS increase, while the optimal area distributions decrease.
(3) With an increase in the HT coefficients of the hot HEX and cold HEX, the double-maximum POW and the optimal temperature of the LTHS increase, while the optimal area distributions and the corresponding TEF under the double-maximum POW decrease. With an increase in the total HT area of the HEXs, the double-maximum POW, the optimal area distributions and the corresponding TEF under the double-maximum POW increase, while the optimal temperature of the LTHS decreases.
(4) When the HT coefficients of the hot HEX and cold HXE are different, it will have a greater impact on the POW and the optimal area distributions of the HEXs. With an increase in the HT coefficient of the cold HEX, the double-maximum POW, the optimal area distribution of the hot HEX and the optimal temperature of the LTHS increase, while the optimal area distribution of the cold HEX and the corresponding TEF under the double-maximum POW decrease. When the HT coefficients of the hot HEX and cold HEX are the same, the changes in the optimal area distributions of the hot HEX and cold HEX are the same.

## Abbreviations

CBC	Closed Brayton cycle
ECHE	Endoreversible Carnot heat engine
FTT	Finite time thermodynamics
HEX	Heat exchanger
HT	Heat transfer
LTSH	Low-temperature heat sink
POW	Power output
TEF	Thermal efficiency
WF	Working fluid
FPDT	Finite Physical Dimensions Thermodynamics
**Nomenclature**	
$A_{r}$	Area of radiation surface ($m^{2}$)
$F_{1}$	Area of hot heat exchangers ($m^{2}$)
$F_{2}$	Area of cold heat exchangers ($m^{2}$)
$K_{1}$	Heat transfer coefficient of hot heat exchanger ($W/\left(m^{2}\cdot K\right)$)
$K_{2}$	Heat transfer coefficient of cold heat exchanger ($W/\left(m^{2}\cdot K\right)$)
P	Power output (W)
$Q_{1}$	heat flux rate of hot side (W)
$Q_{2}$	heat flux rate of cold side (W)
$Q_{3}$	heat flux rate of radiator panel (W)
T	Temperature (K)
**Greek Letters**	
ε	Emissivity of the radiator (-)
η	Thermal efficiency (-)
$\eta_{f}$	Fin efficiency (-)
σ	Boltzmann constant ($W/(m^{2}\cdot K^{4})$)
**Superscripts**	
H	Temperature of the high-temperature heat source
h	Temperature of the high-temperature work fluid
L	Temperature of the low-temperature heat sink
l	Temperature of the low-temperature work fluid
max	Maximum value
max,max	Double maximum value
opt	Optimum
0	Environment
1−4	Cycle state points
